# Association of Selenium Intake and Selenium Concentrations with Risk of Type 2 Diabetes in Adults: A Narrative Review

**DOI:** 10.3390/metabo13060767

**Published:** 2023-06-19

**Authors:** Maha Alharithy, Nora Alafif

**Affiliations:** Department of Community Health Sciences, College of Applied Medical Sciences, King Saud University, Riyadh 11433, Saudi Arabia

**Keywords:** selenium, selenium intake, adults, young adult, type 2 diabetes, insulin resistance, glycosylated hemoglobin, blood glucose

## Abstract

Several recent studies have suggested selenium (Se) as a potential risk factor for diabetes mellitus (DM); however, the relationship between high Se levels and type 2 diabetes mellitus (T2DM) risk remains unclear. This review article aimed to provide a comprehensive discussion to clarify the association between high dietary Se intake and blood Se concentrations and the risk of T2DM among adults. We conducted searches in the PubMed, Science Direct, and Google Scholar databases for the years 2016 to 2022 and evaluated 12 articles from systematic reviews, meta-analyses, cohort studies, and cross-sectional studies. This review found a controversial association between high blood Se concentrations and T2DM risk while demonstrating a positive correlation with DM risk. In contrast, there are conflicting results regarding the association between high dietary Se intake and T2DM risk. Thus, longitudinal studies and randomized controlled trials are needed to better elucidate the link.

## 1. Introduction

Diabetes mellitus (DM) is a global burden linked to mortality and morbidity [1]. According to the International Diabetes Federation (IDF), DM globally is predicted to rise to 783 million adults by 2045—a number that has increased from 537 million in 2021 [2]. In Saudi Arabia, the World Health Organization (WHO) estimates that around 7 million individuals suffer from DM [3]. Furthermore, almost 3 million people in Saudi Arabia are at risk of developing type 2 diabetes mellitus (T2DM) [4]. Globally, over 462 million people had T2DM in 2017, according to the Global Burden of Disease report [5,6]. Locally, the rate of T2DM incidence was 16.4%, and this finding is consistent with those of the IDF: Saudi Arabia has been identified as one of the top ten countries with the highest prevalence of T2DM [7,8].

DM is a chronic metabolic disease that has two main types: type 1 diabetes mellitus (T1DM) and T2DM [2]. T1DM is typically associated with B-cell destruction, leading to absolute insulin deficiency [2]. Conversely, T2DM is a chronic disease characterized by insulin resistance, insulin insufficiency, and insulin secretory dysfunction [2]. DM risk factors include family history, weight gain, inactivity, race, age, gestational diabetes mellitus (GDM), and imbalances in trace elements [9,10]. It was diagnosed with symptoms that include excessive urination, excessive thirst, excessive hunger, weight loss, and blurred vision [11], a high fasting blood glucose (FBG) level, and high hemoglobin A1c (HbA1c) lab results [12]. Moreover, hyperglycemia is a major characteristic of DM complications, which leads to imbalances in trace elements, and increased production of reactive oxygen species leads to an increase in oxidative stress, which impacts the progression of T2DM [10,13].

Selenium (Se) is an essential nutrient that is naturally present in the human body in an organic form called selenoproteins, which serve various bodily functions, such as acting as an antioxidant, anti-inflammatory, and regulating thyroid hormones and glucose metabolism [14,15]. Se can be found in plant foods and animal products, including seafood, meat, poultry, eggs, dairy products, bread, cereals, and other grain products [16]. The recommended daily amount of Se for adults is 55 µg, and the best way to ensure adequate Se intake is by eating a variety of foods [16]. There is concern that high exposure to Se may be associated with the risk of DM or insulin resistance [17]. Se has recently come to light as a factor in the etiology and progression of T2DM risk [18,19]. As a result, the cell redox balance is deregulated and can impair the chemical interactions that produce an insulin signaling cascade [20]. Consequently, overexpression of selenium-dependent antioxidant enzymes reduces intracellular reactive oxygen species and dysregulates insulin synthesis, insulin secretion, and key regulators of β-cells [21]. Oxidative stress plays a significant role in insulin resistance and T2DM, depending on dietary Se intake and basal Se plasma concentrations [17].

Researchers have been studying the correlation between high Se intake and the risk of DM, or insulin resistance, based on the results of observational studies. A cross-sectional study conducted on adults in China showed a positive association between high dietary Se and the incidence of T2DM [17]. A recent systematic review and dose response meta-analysis of nonexperimental studies found that high levels of Se exposure were associated with an increased risk of T2DM [22]. Another study found that higher Se intake was also associated with increased plasma glucose levels and HbA1c [23]. In contrast, two studies showed that there was a significant association between the risk of T2DM and both high and low dietary Se intake [24,25]. However, another cross-sectional study published in 2021 based on a two-year cohort study found no significant differences in body mass index (BMI), smoking status, or prevalence of T2DM across Se intake levels [26]. Despite these recent findings, the relationship between Se levels and T2DM risk is still unclear, raising concern that high Se levels may lead to more cases of T2DM. To fill this gap, we aimed to present a comprehensive discussion to clarify the association of high dietary Se intake and blood Se concentrations with T2DM risk among adults.

## 2. Method

### 2.1. Review Question and Population, Exposure, Comparison, and Outcome (PECO) Statement

We conducted this review to provide a comprehensive discussion to clarify the association between high dietary Se intake and blood Se concentrations and the risk of T2DM among adults. We used the PECO statement for the search strategy, and it is described in more detail in Table 1 below.

### 2.2. Search Strategy

The literature search for this review was conducted through three databases: PubMed, Science Direct, and Google Scholar. The included studies were published from 2016 to 2022. We searched using the following combination of search terms, (((((((((Dietary Selenium) OR (Selenium Intake)) OR (Selenium)) AND (Adults)) OR (Young Adult)) AND (Type 2 diabetes)) OR (Insulin resistance)) OR (Hyperglycemia)) AND (Glycosylated hemoglobin)) AND (Blood glucose).

### 2.3. Study Eligibility Criteria

#### 2.3.1. Inclusion Criteria

The inclusion criteria were (a) quantitative studies and English-language studies of adults; (b) studies that involved exposure to dietary Se intake, blood Se concentration, or both; and (c) studies that used HbA1C, FBG, and other T2DM risk markers to measure outcome.

#### 2.3.2. Exclusion Criteria

The exclusion criteria were (a) qualitative studies and reviews; (b) studies with animals or Se supplementation; and (c) studies conducted on children or the elderly.

### 2.4. Data Collection and Analysis

#### Data Extraction

Data were extracted from the eligible studies, including the aim of the study, authors, year, study population, country, study design, matrix, and results. These data are displayed in Tables 2–4.

## 3. Results and Discussion

### 3.1. Results of the Search

**Figure 1:**
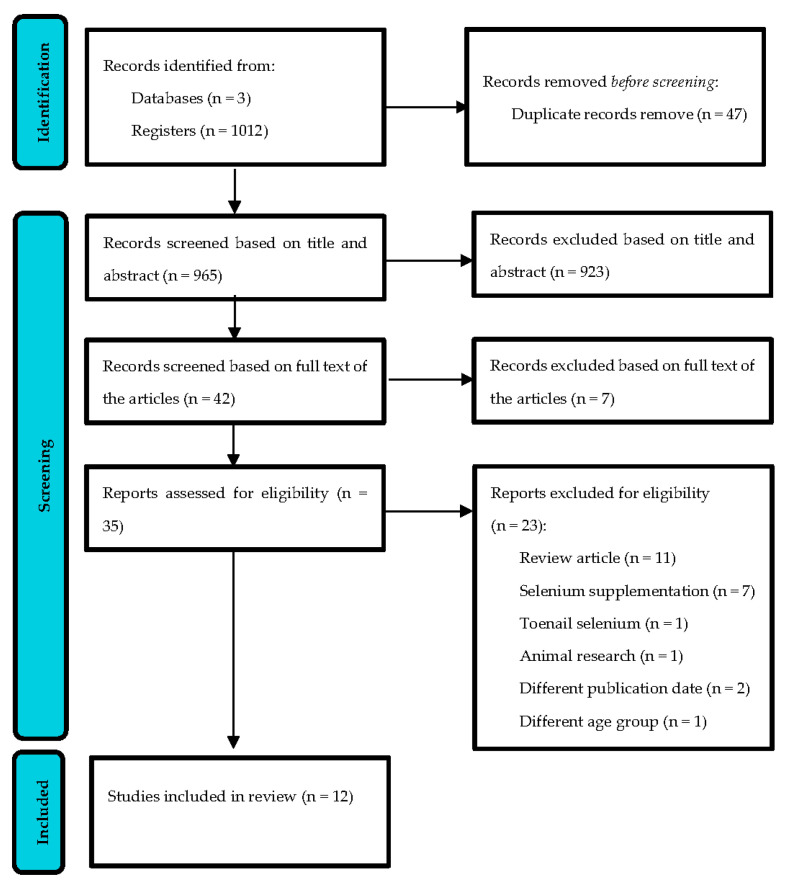
Flow diagram.

As shown in Figure 1, a total of 1012 articles were identified using the defined search strategy. After the removal of 47 duplicate articles, 965 articles were screened for eligibility through a review of the titles and abstracts. After screening, the full text of the remaining 42 articles was reviewed, and 35 potential studies were identified for inclusion. After reviewing the full text of those articles, 12 studies were found eligible for inclusion in this review. These are described in more detail in Table 1, Table 2 and Table 3.

### 3.2. Characteristics of Included Trials

#### 3.2.1. Countries, Sample Sizes and Genders

Five of the reviewed studies were conducted in China [17,23,27,28,29], two in South Korea [13,30], one in Qatar [31], one in Algeria [24], one in Brazil [26], one in Australia [32], and one in Norway [33]. The participant sample sizes ranged from 290 to 34,339 [23,24]. Both males and females were considered in all studies except one, which included only female participants [24]. According to categorization, four studies groped participants into an experimental group consisting of individuals with DM and a control group consisting of those without DM [23,30,32,33], six studies groped participants into a T2DM group and a control group [17,24,26,27,28,29], and two studies analyzed the results without any comparisons of the study population [13,31].

#### 3.2.2. A Methodological Comparison of Tools Used to Detect the Association between High Dietary Se Intake and Blood Se Concentrations with the Risk of T2DM

##### Dietary Se Intake and Risk of T2DM

Overall, five studies measured the association between dietary Se intake and the risk of T2DM [17,24,26,27,31]. The studies used various dietary assessment tools such as interviews, 24-h dietary recalls [27,31], food frequency questionnaires [17,26], or 72-h dietary recalls [24]. Some studies used some methods to increase the accuracy of dietary assessment tools, such as repeating the 24-h dietary recall for two non-consecutive days and taking the mean food intake of the two days [31] or repeating the 24-h recalls for three consecutive days and using food-weighing methods [24]. The 24-h dietary recall was the most commonly used tool in the studies to measure the dietary Se intake [27,31]. Additionally, these studies measure the risk of T2DM using different DM risk markers such as FBG [24,27,31], HbA1c [17,24,27,31], HOMA-IR [17], two-hour plasma glucose levels from an oral glucose tolerance test (OGTT) [31], or self-reported doctor-diagnosed DM [26].

##### Blood Se Concentration and Risk of T2DM

Five studies measured the association between blood Se concentration and the risk of T2DM [13,28,29,32,33]. All the studies used serum Se concentrations [13,28,29,32] and only one study used fasting plasma metal concentrations to measure blood Se concentrations [33]. In addition, these studies measure the risk of DM by using FBG [28,29,32], HbA1c [13,28,29,32], HOMA-IR [32], fasting plasma insulin [32], C-peptide and anti-glutamic acid decarboxylase (GAD) antibodies or use of anti-diabetic medications [28], or self-reported doctor-diagnosed DM [13,28,33]. For patients without a known DM diagnosis, measure FINDRISC and undergo an OGTT [33]. FBG [28,29,32], HbA1c [13,28,29,32] and DM presence [13,28,33] were the most common DM risk markers used in previous studies.

##### Association of Dietary Se Intake and Blood Se Levels with Risk of T2DM

Two studies measured the association between dietary Se intake and blood Se levels with the risk of DM [23,30]. The systematic review of the meta-analysis study measured the dietary Se intake using a food diary survey and the blood Se using serum Se concentration [30]. Furthermore, the risk of DM was measured according to the presence of DM in participants [30].

Moreover, a cross-sectional study measured dietary Se using 24-h recalls by two interviewers and measured blood Se by serum Se concentrations [23]. The risk of DM was measured using FBG, HbA1c, and self-reported doctor-diagnosed DM [23].

### 3.3. Epidemiology of the Relationship between Se and Risk of T2DM

To assess the relationship between Se levels and T2DM risk, we sorted the reviewed studies according to the Se interventions measured, such as dietary Se intake or blood Se concentrations, and the associated risk of T2DM.

#### 3.3.1. Dietary Se Intake and Risk of T2DM

We reviewed five observational studies, including cross-sectional and cohort designs, that have investigated the association between dietary Se intake and the risk of T2DM [17,24,26,27,31]. Despite all five studies finding an association, the results differed. These are described in more detail in Table 2.

Some studies revealed a positive correlation between dietary Se intake and the risk of T2DM with OR and CI values of 1.44 (95% CI: 1.09, 1.89), 1.66 (95% CI: 1.38, 1.99), and 2.21 (95% CI: 1.06, 4.38) [17,24,31]. A cross-sectional study conducted in 2021 used the National Health and Nutrition Examination Surveys from 2003 to 2014 to investigate the correlation between Se intake and DM in general rather than only T2DM specifically [31]. The study included six survey cycles and follow-ups with nearly 19,000 people across a mean of 6.6 years [31]. The results demonstrated a significant positive association between Se intake and DM [31]. A later cross-sectional analysis of 8824 North Chinese adults revealed a significant linear association between high dietary Se intake of 52.43 μg per day and an increased risk of T2DM of 20.4% in the studied population [17]. Conversely, a study conducted in Algeria with a sample population of 290 females compared a control group with a group of participants with T2DM, and no confounding factors were identified [24]. The researchers discovered a significant U-shaped relationship between T2DM risk and dietary Se intake, both below and above the dietary recommendations [24].

In contrast, two observational studies found a significant negative relationship between Se and the risk of T2DM [26,27]. The cross-sectional study based on the Cohort of Universities of Minas Gerais, Brazil (CUME) project looked at dietary Se intake and the risk of T2DM in 4106 Brazilians [26]. The researchers concluded that Se intake did not affect T2DM prevalence, despite the high mean Se intake (157.4 μg/day) of the sample group [26]. Another cohort study found that dietary copper and Se intakes were not linked with T2DM risk in Chinese people from 15 provinces over the 9.2 years of follow-up with a large population [27]. However, dietary Se intake significantly affected the link between dietary copper intake and T2DM risk, and there was a significant interaction between dietary copper and Se intakes on the risk of T2DM [27].

**Table 2:** Summary of reviewed studies on the association of selenium (Se) intake and type 2 diabetes (T2DM) risk.

Title	Author, Year, Country	Study Design	Matrix/Mean Se	Aim	Study Population/Sex (Men, Women)/Mean Age	Confounding Factor	Statistical Analysis	Result
Association between selenium intake, diabetes, and mortality in adults: findings from National Health and Nutrition Examination Survey (NHANES) 2003–2014	Hoque and Shi, 2021. Qatar [31]	Cross-sectional study	Dietary Se intake (70.60) μg/day	To investigate the association between Se intake, diabetes, all-cause and cause-specific mortality in a representative sample of US adults	18,932 adults (M: 1264, W: 3472) based on the National Health and Nutrition Examination Survey (NHANES) 2003 to 2014. Mean Age: 53·7 years old	Age, BMI, sex, race, education level, leisure time, physical activity, smoking status, and alcohol intake.	OR = 1.44 (95% CI: 1.09, 1.89)	High Se intake was positively associated with a higher prevalence of risk DM after adjusting for the confounding factors.
Dietary selenium intake and risk of type 2 diabetes in a population of western Algeria	Siddiqi et al., 2020. China [17]	Cross-sectional study	Dietary Se intake (52.43) μg/day	To investigate the significance of dietary Se and T2DM in North Chinese adults.	8824 adults (M: 3000, W: 5824) based on the Harbin Cohort Study on Diet, Nutrition, and Chronic Noncommunicable Diseases (HDNNCDS). Mean Age: 49.74 years old	Age, sex, BMI, waist, smoking, alcohol intake, energy intake, body fat, education, family history of diabetes, exercise, hypertension, and coronary heart disease.	OR: 1.66 (95% CI: 1.38, 1.99); *p* < 0.005)	High Se intake was positively associated with higher FBG, HbA1c, and risk of T2DM after adjusting for the confounding factors.
The Correlation between Dietary Selenium Intake and Type 2 Diabetes: A Cross-Sectional Population-Based Study on North Chinese Adults	Behar et al., 2020. Algeria [24]	Cross-sectional study	Dietary Se intake (72.40–70.30) μg/day in diabetics controls respectively	To investigate the association between dietary selenium intake and the risk of type 2 diabetes in a female population from western Algeria.	290 adults (W: 290) based on Public Proximity Medicine Establishment of Remchi, Algeria. Mean Age: 59.20 years old	None	High Se intake OR: 2.21 (95% CI: 1.06, 4.38; *p* = 0.036). While the low Se intake OR: 2.52 (95% CI: 1.25, 5.09; *p* = 0.010)	High and low Se intake were positively associated with higher FBG, HbA1c, and with a risk of T2DM.
Dietary Selenium Intake and Type-2 Diabetes: A Cross-Sectional Population-Based Study on CUME Project	Dias et al., 2021. Brazil [26]	Cross-sectional study	Dietary Se intake among men (148.9 μg/day) and among women (131.4 μg/day).	To investigate the association between dietary Se and type-2 diabetes (T2DM) in the Brazilian cohort [Cohort of Universities of Minas Gerais (CUME)].	4106 adults (M:1209, W: 2807) based on the Brazilian cohort (CUME) project. Mean Age: 42 years old	Gender, age, BMI, smoking status, alcoholic intake, physical activity, energy intake, sugar, carbohydrates, lipids, fiber, and energy-adjusted meat intake.	Hierarchical cluster analysis. *p* = 0.936	High Se intake was not associated with a greater risk of T2DM after adjusting for the confounding factors.
Dietary Copper and Selenium Intakes and the Risk of Type 2 Diabetes Mellitus: Findings from the China Health and Nutrition Survey	Cui et al., 2022. China [27]	Cohort study	Dietary Cu or Se intakes (41.1) μg/day	To examine the prospective associations between dietary Cu and Se intakes and T2DM risk in Chinese adults	14,711 (M: 7333, W: 7378) adults based on China Health and Nutrition Survey (CHNS) 1997 to 2015. Mean Age: 45 years old	Sex, age, BMI province of residence, educational level, alcohol intake, smoking status, physical activity, urbanization index, baseline hypertension, dietary variables, including (total energy intake, cholesterol, fiber, dietary glycemic index, ratio of animal protein to plant protein, and ratio of polyunsaturated fatty acids to Saturated fatty acids).	Time-dependent Cox proportional hazard regression *p* = 0.82	High Se intake was not associated with an increased risk of T2DM when fully adjusted for the confounding factors.

#### 3.3.2. Blood Se Concentration and Risk of T2DM

Most of the studies, including a cohort study and cross-sectional studies, showed a positive association between high blood Se concentration and risk of DM, with OR and (Cl) values of 1.12 (95% CI: 1.06, 1.18) for each 10 μg/L increment in Se levels, 1.14 (95% CI: 0.99, 1.30), and 1.12 (95% CI: 1.01, 1.24; *p* = 0.0270) for each 10 μg/L increment in Se levels [13,28,29]. These are described in more detail in Table 3.

A prospective cohort study of a large Chinese population investigated the associations between internal exposures to multiple metals and DM risk. Significant associations were found with Se, titanium, arsenic, and antimony levels (*p* < 0.01) [28]. In contrast, no significant associations were found for other metals [28]. Another study that recruited a population from the National Health and Nutrition Examination Survey in the United States between 2011 and 2014 demonstrated that an increase of 10 μg/L in serum Se increased the prevalence of DM by 12% [13]. Researchers examined 2903 patients for a possible relationship between DM and high serum Se by measuring HbA1c and FBG [29]. A positive association with the odds of DM was found, but Se was independently associated with DM only in females younger than 65 years old [29]. Conversely, a study conducted in 2021 analyzed the association of Se concentration with DM markers such as HbA1c, fasting plasma insulin, and insulin resistance estimated via HOMA-IR [32]. After adjusting for confounding factors, no significant relationship was found between Se and DM prevalence [32].

An additional study showed a positive relationship between Se concentration and T2DM specifically. A nonlinear dose response meta-analysis of five studies of serum Se levels suggested that low serum Se levels (97.5 μg /L) and high serum Se levels (>132.5 μg /L) are both significantly associated with the prevalence of T2DM (*p* = 0.033) [25]. That indicates a U-shaped relationship between Se and T2DM [25]. However, a study based on the Nord-Trøndelag Health Study (NTNU) survey investigated the relationship between the levels of 25 blood trace elements and T2DM prevalence [33] and found a significant association between higher boron, calcium, and silver levels and T2DM prevalence, while Se levels were negatively correlated with T2DM [33].

**Table 3:** Summary of reviewed studies on the association of blood Se level and T2DM risk.

Title	Author, Year, Country.	Study Design	Matrix/Mean Se	Aim	Study Population/Sex/Age	Confounding Factor	Statistical Analysis	Result
The Association of Circulating Selenium Concentrations with Diabetes Mellitus	Liao et al., 2020. China [29]	Cross-sectional study	Blood Se concentrations (136.4 ± 19.60) μg/L	To analyze the association of circulating selenium level with DM, and further explore their relationship through sex and age subgroups.	2903 (M: 1431, W: 1472) adults based on the National Health and Nutrition Examination Survey (NHANES) 1999–2006 Mean Age: 61.9 years old	Age, sex, BMI, systolic blood pressure, Total cholesterol, triglyceride, low-density lipoprotein cholesterol, C-reactive protein, smoking, race, and hypertension	OR: 1.12 (95% CI: 1.01, 1.24; *p* = 0.0270) for each 10 μg/L increment in Se levels.	High Se concentration was positively associated with a greater risk of DM with significant differences based on sex and age after adjusting for confounding factors.
Association between serum selenium level and the prevalence of diabetes mellitus in U.S. population	Moon et al., 2019. South Korea [13]	Cross-sectional study	Serum Se concentrations (129) μg/L	To ascertain the relationship between selenium and DM.	3406 adults (M: 1675, W: 1731) based on the NHANES 2011 to 2014. Mean Age: 46 years old	Age, sex, race/ethnicity, hypertension, dyslipidemia and BMI	OR: 1.12; (95% CI: 1.06, 1.18) for each 10 μg/L increment in Se levels.	Higher serum Se concentration was positively associated with a greater risk of DM after adjusting for the confounding factors.
Selenium Status Is Associated with Insulin Resistance Markers in Adults: Findings From the 2013 to 2018 National Health and Nutrition Examination Survey (NHANES)	Cardoso et al., 2021. Australia [32]	Cross-sectional study	Blood Se concentrations (196.2) μg/L	To investigate the association between blood selenium concentration and glucose markers in a representative sample of the US population, which is known to have moderate to high exposure to selenium	4339 (M: 2097, W: 2242) adults based on the NHANES 2013 to 2018 Mean Age: 47.3 years old	Age, sex, smoking status, physical activity, BMI, and metabolic syndrome.	Mean difference: 3.9 μg/L, (95% CI: 8.8, 0.85, *p* = 0.104)	High Se concentration was not associated with a higher risk of DM after adjusting for the confounding factors.
Trace Element Status in Patients with Type 2 Diabetes in Norway: The HUNT3 Survey	Simic et al., 2017. Norway [33]	Cross-sectional study	Blood Se concentrations (102.3) μg/L	To investigate the association between prevalent T2DM and the concentrations of 25 trace elements in whole blood, and the relationships between T2DM duration and blood levels of the trace elements that we found to be related to T2DM prevalence.	876 (M: 434, W: 442) adults based on population from Nord-Trondelag Health Study (HUNT3 Survey). Mean Age: 62.3 years old	Age, sex, smoking status, alcohol consumption, fat fish and milk intake, family history of DM, weight, height, waist and hip circumference, education level, ongoing pregnancy, and income	OR: 1.13 (95% CI: 0.65, 1.96) *p* = 0.367.	High Se concentration was not associated with a higher risk of DM after adjusting for the confounding factors.
Associations of multiple plasma metals with incident type 2 diabetes in Chinese adults: The Dongfeng-Tongji Cohort	Yuan et al., 2018. China [28]	Cohort study	Serum Se concentrations (63.01) μg/L	To examine the relationship between plasma concentrations of 23 metals and the incidence of T2DM among Chinese senior adults.	2078 (M: 928, W: 1150) adults based on the Dongfeng–Tongji (DFTJ) cohort study 2008 to 2010. Mean Age: 63.87 years old	BMI, smoking and alcohol intake, education level, physical activity, family history of diabetes, baseline history of hypertension and hyperlipidemia, and baseline eGFR levels.	OR: 1.14 (95% CI: 0.99, 1.30)	High Se intake was positively associated with a greater risk of T2DM after adjusting for the confounding factors.

#### 3.3.3. Association of Dietary Se Intake and Blood Se Levels with Risk of T2DM

Systematic review meta-analyses and cross-sectional studies have demonstrated a remarkable positive association between high dietary Se intake and blood Se levels and the risk of DM, with OR and CI values of 2.139 (95% CI: 1.763, 2.596) and 1.61 (95% CI: 1.10, 2.36), respectively [23,30]. These are described in more detail in Table 4. However, no studies included in our review investigated this relationship regarding T2DM risk.

A meta-analysis of observational studies that included 20 research papers (17 cross-sectional and 3 cohort studies) showed that high levels of Se were significantly related to the presence of DM. Furthermore, a significant positive relationship was found between DM and Se levels that were measured in the blood, diet, and urine studies, in contrast to studies measuring Se in nails [30]. In addition, Lin and Shen evaluated the association of both Se intake and serum concentration levels with DM risk in 34,940 subjects from China. The mean Se intake and serum Se concentration were 98 ± 55 μg/day and 129 ± 22 ng/mL, respectively. The study showed that high dietary Se intake and serum Se levels were positively associated with elevated plasma glucose and HbA1c levels [23].

## 4. Conclusions

The association between Se and the risk of T2DM is obviously complicated. This mini review illustrated the complex results showing the relationship between Se and T2DM risk in systematic reviews, meta-analyses, cohort studies, and cross-sectional studies.

This mini-review found that most of the investigated studies examined the association between high blood Se levels and the risk of DM disease; these studies illustrated a positive association between the two. Some studies have revealed a controversial correlation between high blood Se concentrations and T2DM risk. In contrast, there are conflicting results regarding the association between high dietary Se intake and T2DM risk.

The main strength of this mini-review is that it is the first contemporary review of the relationship between dietary Se intake and blood Se concentration levels and the risk of T2DM. It included a large population. However, a limited number of studies have investigated the association of high dietary Se and Se concentrations with T2DM risk. Moreover, most review studies are cross-sectional evaluations, which cannot explain causal relationships in outcomes. The range of dietary Se intake or blood Se concentration levels and the tools used to measure the outcomes and exposure are inconsistent in the reviewed studies. Finally, there was variability in terms of the studies’ geographical area. In future research, further randomized controlled trials studying dietary Se and both dietary Se intake and blood Se concentration are necessary to confirm these findings. Furthermore, longitudinal studies using standard measures of outcomes will validate those findings.

## Figures and Tables

**Table 1:** The PECO statement used for the search strategy.

Acronym	Definition	Description
P	Population	Adults
E	Exposure	Dietary Se intake and/or Se concentrations
C	Comparison	-
O	Outcome	HbA1c, FBG, and other T2DM risk markers

**Table 4:** Summary of reviewed studies on the association between Se intake, blood Se level, and T2DM risk.

Title	Author, Year, Country	Study Design	Matrix/Mean Se	Aim	Study Population/Sex/Age	Confounding Factor	Statistical Analysis	Result
Association of dietary and serum selenium concentrations with glucose level and risk of diabetes mellitus: A cross sectional study of national health and nutrition examination survey, 1999–2006	Lin et al., 2021. China [23]	Cross-sectional study	Dietary Se intake (55 μg/d) and serum Se concentration (129 ng/mL)	To examine whether dietary selenium intake and the serum selenium concentration are associated with plasma glucose and glycosylated hemoglobin levels and diabetes risk in participants from the United States.	34,940 (M: 17,024, W: 17,916) adults based on the National Health and Nutrition Examination Survey (NHANES) 1999–2006. Mean Age: 30.32 years old	Age, sex, race, education, BMI, smoking, hypertension, alcohol, intake, energy intake, total fat and dietary fiber.	OR: 2.139, (95% CI: 1.763, 2.596, *p* < 0.001)	High Se intake and high serum Se concentration were positively associated with higher plasma glucose, HbA1c, and the risk of DM after adjusting for the confounding factors.
Association between Serum Selenium Level and the Presence of Diabetes Mellitus: A Meta-Analysis of Observational Studies	Kim et al., 2019. South Korea [30]	Systematic review-meta-analysis	Se levels in diet, blood, urine, and nails Cut-off value of Se level of all group: <100 μg/L, 100–120μg/L, >120 μg/L	To perform a comprehensive meta-analysis to clarify the impact of Se on DM.	47,930 adults were enrolled, and 6347 of them had DM. Sample sizes of these studies ranged from 128 to 8876 participants.	None	OR: 1.61; (95% CI: 1.10, 2.36)	High Se intake and blood, and urine levels were positively associated with a greater risk of DM.
